# Relapse of cervical tuberculous lymphadenitis immediately after completion of effective anti‐tuberculosis treatments

**DOI:** 10.1002/rcr2.555

**Published:** 2020-04-07

**Authors:** Yuya Kimura, Masahiro Shimada, Masahiro Kawashima, Akira Yamane, Hideaki Nagai, Hirotoshi Matsui

**Affiliations:** ^1^ Center for Pulmonary Diseases National Hospital Organization Tokyo National Hospital Tokyo Japan

**Keywords:** Cervical tuberculous lymphadenitis, paradoxical reaction, relapse, surgical excision

## Abstract

Most cases of lymph node enlargement after completing tuberculosis (TB) treatment are due to paradoxical reaction (PR), not relapse, and therefore, do not require re‐treatment. We herein report a case of a 28‐year‐old man who had developed cervical TB lymphadenitis and exhibited re‐enlargement of the same lymph nodes one month after completing effective TB chemotherapy, which was microbiologically proven as relapse. The patient noticed painful cervical lymphadenopathy one month after completion of chemotherapy for TB lymphadenitis. Combination chemotherapy with multiple anti‐TB drugs was resumed with suspicion of relapse. But, with his symptoms having worsened, surgical excision was performed. *Mycobacterium tuberculosis* was cultured from the dissected lymph nodes. Early regrowth of the lymph nodes after completing treatment can derive from microbiological relapse, in addition to PR. Surgical excision was useful for the microbiological diagnosis of the relapse. We must take care of lymph node re‐enlargement in consideration of TB relapse.

## Graphical abstract

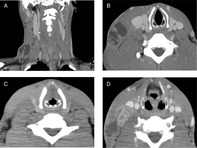

## Introduction

Post‐treatment lymphadenopathy occurred in up to 15% of patients who have completed tuberculosis (TB) chemotherapy 1, 2. In the biggest prospective cohort study for non‐HIV patients having completed TB lymphadenitis treatments, out of 36 patients with post‐treatment lymph node enlargement, 33 (91.7%) improved spontaneously (paradoxical reaction (PR)) and three (8.3%) improved with re‐treatment, although relapses were not proven microbiologically in all the surveyed cases. Their conclusion was post‐treatment lymph node enlargement should be monitored carefully without re‐treatment 2. The differences of the characteristics between PR and relapse cases were not obvious.

As far as we searched, there was no case report of post‐treatment lymphadenopathy caused by microbiologically proven relapse. In this report, we present a patient with lymph node re‐enlargement after treatment, which was proven as relapse by the culture of lymph nodes resected to control pain.

## Case Report

**Figure 1:**
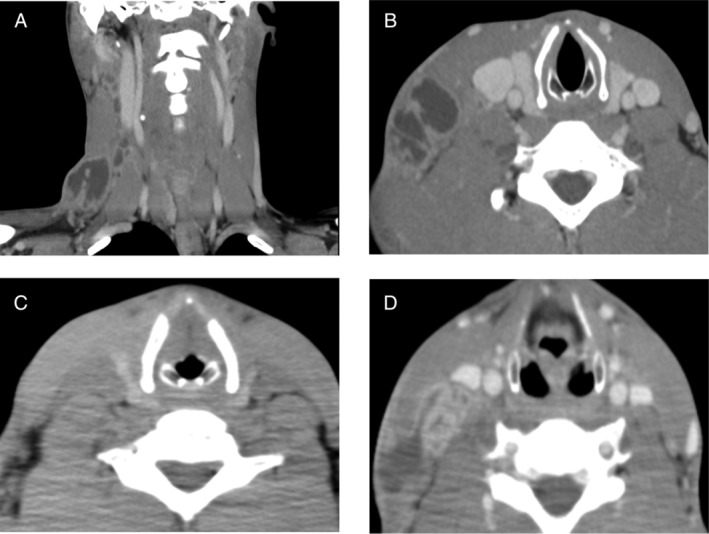
(A, B) Enhanced computed tomography (CT) on the first presentation showed right cervical lymphadenopathy. (C) CT six months after the initiation of anti‐tuberculosis (TB) drugs revealed decrease in the size of the lesions. (D) Enhanced CT obtained one month after treatment completion. Multiple cervical lymphadenopathy was seen in the same place as seen before.

A previously healthy 28‐year‐old Filipino man presented to our hospital with one‐month history of pain and swelling on the right side of his neck and a slight fever. An enhanced computed tomography (CT) scan (Fig. 1A, B) showed right cervical lymphadenopathy and cavity lesion of the left upper lobe. Fine‐needle aspiration (FNA) specimen from a cervical lymph node gave a positive acid‐fast bacilli (AFB) stain and TB‐polymerase chain reaction (PCR) result. With the diagnosis of cervical TB lymphadenitis and pulmonary TB, combination chemotherapy consisting of rifampicin, isoniazid, ethambutol, and pyrazinamide was started.

He was first put on directly observed treatment, short‐course (DOTS) in hospital. Cultures of the FNA specimen and the patient's sputum grew *Mycobacterium tuberculosis* by MGIT (Mycobacteria Growth Incubator Tube; BBL MGIT, Becton, Dickinson and Company, USA) system. The drug susceptibility testing with a Well‐pack® method (one of the modified methods of Ogawa egg slant; Nihon BCG, Japan) revealed the bacilli were sensitive to isoniazid (0.2S), rifampicin (1.0S), and ethambutol (5.0S). As the susceptibility test with an MGIT system identified isoniazid (0.1R) and pyrazinamide resistance (100R), pyrazinamide was discontinued and levofloxacin was added to the regimen, considering the possibility of resistance. Ethambutol was also discontinued after two months of usage. After discharge, he was checked week by week with a public health nurse under DOTS of local public health centre. Finally, isoniazid, rifampicin, and levofloxacin were continued for a total treatment period of 12 months.

A follow‐up CT scan (Fig. 1C) taken six months after treatment initiation revealed decrease in the size of both cervical and pulmonary lesions. There was no palpable lymph node swelling on his neck at the end of the treatment.

But one month after, he began noticing swelling and pain on the right side of his neck again. An enhanced CT scan (Fig. 1D) revealed multiple cervical lymphadenopathy in the same place as seen before, and on the other hand, improvement of the cavitary lesion in the left upper lobe. FNA of the cervical lymph node, performed again, gave a positive AFB stain and a positive TB‐PCR result. The FNA specimen was so purulent that treatment with the combination of five anti‐TB drugs, that is, rifampicin, isoniazid, ethambutol, pyrazinamide, and levofloxacin, was started in suspicion of TB relapse. Cultures of the FNA specimen and the patient's sputum grew no microbes. As his neck swelling and pain gradually worsened in spite of the re‐treatment, surgical excision of his right lymph nodes was performed three months after the initiation of the re‐treatment. Cultures of the dissected lymph nodes grew *M. tuberculosis* sensitive to all first‐line anti‐TB drugs including pyrazinamide by MGIT system. Relapse was confirmed microbiologically. Anti‐TB drugs were administered for 12 months, consisting of five drugs for two months, followed by four drugs without pyrazinamide until the drug susceptibility testing was known, and rifampicin and isoniazid for the remaining period. The total duration of the chemotherapy was extended to 12 months because cultures of the dissected lymph node were positive at three months after the initiation of chemotherapy. At 12 months after the end of the re‐treatment, there was no re‐enlargement of the lymph nodes.

## Discussion

The clinical course of this patient provided two important clinical suggestions. First, lymphadenopathy in the early stage after completing TB lymphadenitis treatment can derive from microbiological relapse. Second, surgical excision was beneficial to check the presence or absence of live bacilli and to deal with uncontrolled symptoms from lymphadenopathy.

We consider the recurrence of TB of this case as relapse, but not reinfection. Considering that the patient was not immunocompromised and Japan was a country of medium burden of TB with incidence rate of 12.3/100,000 population in 2018, reinfection one month after completing TB treatment would be improbable. Besides, cervical TB lymphadenitis is usually due to reactivation of latent infection, perhaps years earlier, and in rare cases, derive from miliary dissemination to lymph nodes in the setting of primary infection 3, 4. In this case, the patient had finished taking 12 months of anti‐TB drugs one month before the re‐enlargement of cervical lymph nodes. It is difficult to imagine that the primary infection of a strain of TB sensitive to all first‐line anti‐TB drugs would happen. However, we cannot totally deny the possibility of “reinfection” because of absence of DNA analysis like restriction fragment length polymorphisms (RFLPs) for the microbes.

We do not think the relapse occurred due to inadequate TB treatment. We sometimes encounter the discrepancy of drug susceptibility tests between MGIT and Well‐pack. Mitarai et al. reported no difference in the treatment outcome and prognosis between isolates of isoniazid (INH) resistant and INH sensitive by MGIT as far as Ogawa medium (one of the proportional methods) indicated INH sensitive at the concentration of 0.2 μg/mL 5, meaning INH was accountable as an effective drug in our combination treatment.

Most of the post‐treatment lymph node re‐enlargement cases are thought to be caused by PR and does not need reintroduction of anti‐TB drugs. In this case, if we did not perform surgery, it may have been diagnosed with PR, considering the negative culture result of the FNA. We suppose that there were many such misdiagnosed cases due to lack of surgical excision. Although a part of relapse cases may have been spontaneously subsided without anti‐TB drugs, we think re‐treatment is necessary for cases of microbiological recurrence.

### Disclosure Statement

Appropriate written informed consent was obtained for publication of this case report and accompanying images.
